# Navigating Child Oral Health in Western Australia: A Caregiver's Perspective

**DOI:** 10.1111/cch.70095

**Published:** 2025-05-30

**Authors:** Lesley Andrew, Elizabeth Wenden, Mohamed Estai, Ruth Wallace

**Affiliations:** ^1^ School of Nursing and Midwifery Edith Cowan University Joondalup Australia; ^2^ School of Medical and Health Sciences Edith Cowan University Joondalup Australia; ^3^ School of Human Sciences The University of Western Australia Perth Australia

**Keywords:** child, experiences, health promotion, knowledge, oral health, practices, primary caregiver

## Abstract

**Background:**

Early childhood caries (ECC) is the most common chronic health condition among Australian preschool‐aged children. Untreated, ECC can affect children's physical and emotional health and future social opportunities. ECC is largely preventable through primary caregivers' correct understanding of oral health promoting diets, oral hygiene practices and dental service engagement. The study aim is to explore Western Australian (WA) primary caregivers' child oral health knowledge, experience and practices. The Fisher‐Owens socioecological model of child oral health was applied across the study to structure and interpret the findings across the complex, interactive sociocultural influences on child oral health.

**Methods:**

The mixed‐methods convergent research design included a questionnaire and focus group sessions with a purposive sample of primary caregivers of children aged under 5 years across two geographical WA locations (metropolitan and regional areas). Questions sought to understand participant knowledge, practices and experiences across categories of tooth safe diet and feeding practices, oral hygiene and oral health services. Questionnaire data underwent simple descriptive statistical analysis. Focus group data were organised using Nvivo v12 software and analysed using the codebook approach to thematic analysis, in which the coding process is guided by Braun and Clarke's predetermined coding frame. The study explored the social determinants of participants' geographical location, English language status and educational attainment.

**Findings:**

Participants (*n* = 42) completed the questionnaires and attended one of 10 focus groups. Findings reveal that despite the availability of evidence‐based oral health guidelines for health professionals, participants reported they received inconsistent messages on the topic. Widespread confusion is evident from all social backgrounds about child oral health and hygiene, in particular, fluoride use, brushing regimes and dietary sugars. Dental service engagement is suboptimal, influenced by lack of availability, cost and perception of a lack of child‐friendly dental services. Cost is a particular factor influencing lower dental service engagement among English as an additional language primary caregivers, whereas lack of availability affects those in regional areas.

**Recommendations:**

Individual, community and wider structural recommendations are offered to promote equitable, accessible and consistent child oral health promotion and dental services.

**Summary**

- Oral health problems, including caries, are a leading cause of hospital admission in Australian children.
- Early childhood caries in preschool children are highly preventable through correct feeding, oral hygiene and dental engagement practices.
- This study is the first to explore Western Australian primary caregivers' knowledge of correct oral health care practices for their preschool aged children.
- Findings demonstrate widespread confusion and uncertainty of correct oral health care practices among Western Australian primary caregivers across the social determinants of education, geographical location and English Language status.
- Inequity persists in dental service access for families in regional locations and for whom English is an additional language.

## 1 Introduction

Early childhood caries (ECC) is the most common chronic health condition among Australian preschool aged children, affecting around half of this demographic (Australian Institute of Health and Welfare 2023a) and increasing the risk of caries in permanent teeth threefold (Lam et al. 2022). Although EEC data in preschool children are not routinely collected in Western Australia, hospital data reveal potentially preventable dental conditions are the leading cause of hospital admissions in Western Australia (WA) (Australian Institute of Health and Welfare 2023a). Untreated ECC affects children's quality of life, causing acute pain, sepsis and tooth loss that impacts feeding and speech development (Kandelman et al. 2008). ECC may also affect children's self‐esteem and disrupt education, future educational attainment and employment prospects. Tooth loss in particular can compromise children's nutritional status increasing their risk of chronic disease in later‐life (Lam et al. 2022). The impact of ECC extends to family financial and emotional health (Singh et al. 2020).

The National Oral Health Plan 2015–2024 (Australian Government Department of Health and Aged Care 2016) aims to improve oral health status across the Australian population. A key strategy to achieve this goal is ‘broadening the availability of evidence‐based oral health promotion programmes’ (Australian Institute of Health and Welfare 2023a). Substantial evidence demonstrates children's oral health is strongly influenced by primary caregivers' knowledge and understanding of oral health promoting diets, oral hygiene practices and dental service engagement (Baskaradoss 2018; Bridges et al. 2014; Phantumvanit et al. 2018). Currently, however, little is known about primary caregiver oral health knowledge, practice and experiences in Australia with available studies revealing a concerning picture (Andrew et al. 2021). A study of Victorian Australians (Martin‐Kerry et al. 2020), for example, found the early introduction of sugar‐rich cariogenic foods into children's diets is common practice, and brushing of children's teeth is suboptimal (Trinh et al. 2022) as is routine dentist service engagement (Kilpatrick et al. 2012).

A study of primary caregiver knowledge, experiences and practices around child oral health requires an exploration of influential social determinants to ensure useful, authentic findings. In Australia, ECC disproportionately affects disadvantaged and marginalised groups, including low socio‐economic status groups and rural and regional communities (Australian Government Department of Health and Aged Care 2016). Across Australia, approximately 30% of the population live in regional and remote areas including 3% in remote communities (Australian Government Department of Health and Aged Care 2016). Within Western Australia, this figure is 21% of the population (Government of Western Australia. Department of Health 2018).

In Western Australia, children living in rural and regional areas have a 65% higher rate of hospitalisation for emergency oral health treatment than children living in metropolitan areas (Australian Government Department of Health and Aged Care 2016). Although school‐aged children are eligible for free dental treatment through the school dental service, access to this service varies in rural and remote settings and the cost of dental services (including preventative treatment) for preschool children may restrict dental access for families on low incomes (Australian Government Department of Health and Aged Care 2016). English as an additional language (EAL) status is associated with lower dental service engagement (Kilpatrick et al. 2012).

Our study is the first to explore WA primary caregivers' knowledge, experience and practices related to the promotion of oral health among children aged under 5 years. Findings are interpreted through a sociocultural lens, encompassing social determinants relevant to the WA oral health context. These findings offer important new evidence to inform preschool child oral health promotion strategies in WA.

### 1.1 Theoretical Framework

**FIGURE 1:**
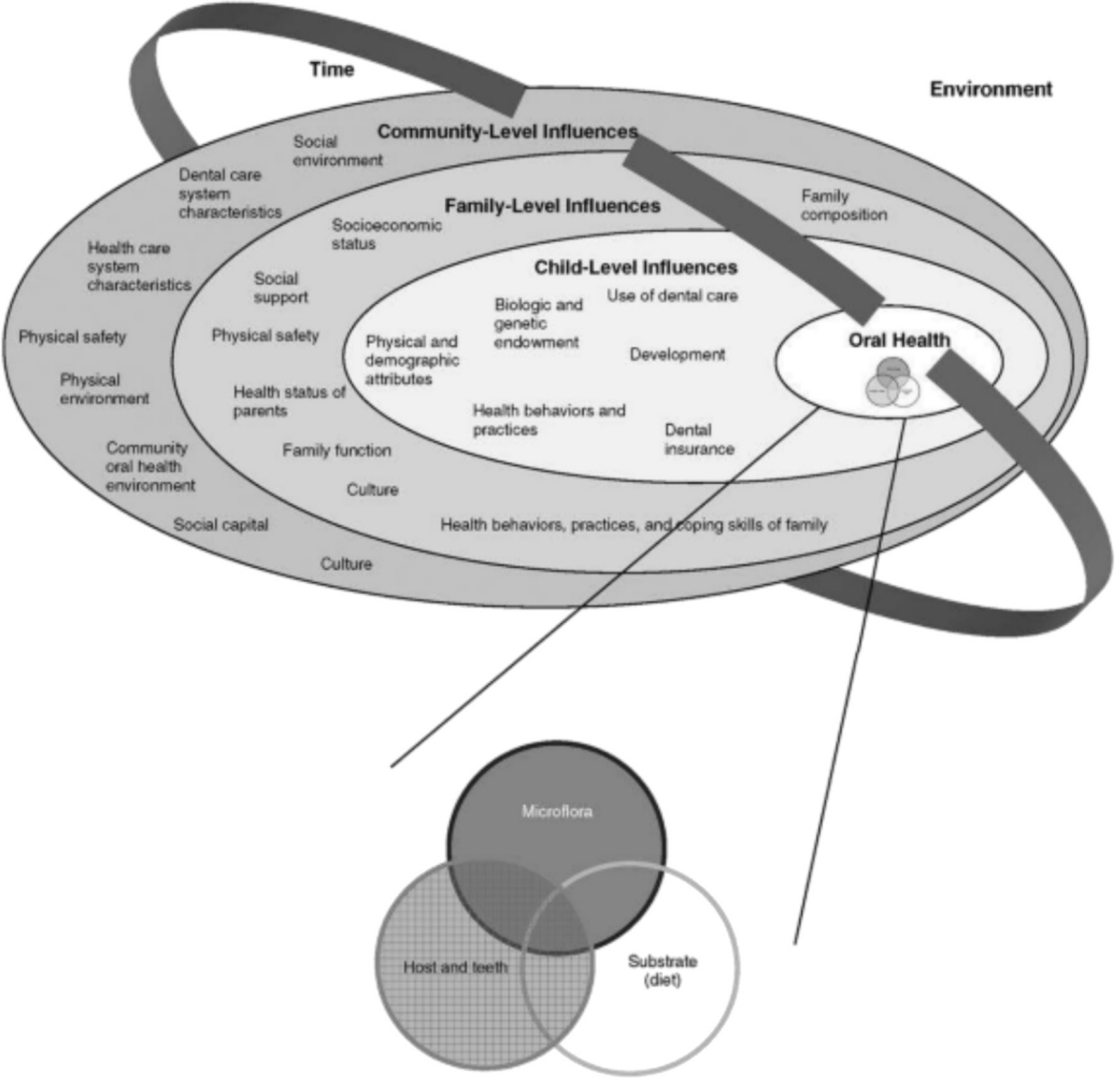
Child, family and community influences on oral health outcomes of children (Fisher‐Owens et al., 2007). *Note:* Twice a day is correct response.

The Fisher‐Owens et al. socioecological model of child oral health (Fisher‐Owens et al. 2007) was developed to inform practitioners of the optimal way to improve children's oral health, which is the key focus of our study. This conceptual model (Figure 1), developed through a health sociology lens, allows us to consider the complex and interactive sociocultural influences on oral health across individual, family and society dimensions within Western Australia. The Fisher‐Owens model uses a multilevel ecological perspective to explain the numerous influences on oral health, considering the complex interplay of individual, family and community factors. These include genetic and biological factors, the social and physical environments in which a child lives, health behaviours and access to dental care. The model also embeds time, recognising the development of oral health diseases over the life course. Ultimately, social ecological models recognise that individuals are embedded within larger social systems and that health outcomes are a result of the interactive characteristics of individuals and environments (Bronfenbrenner 1979). This model has also been used to consider the specific maternal role in child caries prevention (Xiao et al. 2019) and child oral health among migrant and refugee populations (Riggs et al. 2014).

In this study, the model is applied to data collection, specifically guiding the types of questions used in the survey and question guide, as well as data analysis, interpretation of findings (Fisher‐Owens et al. 2007) and the development of holistic and practical recommendations.

## 2 Methods

### 2.1 Study Design

The research team consisted of five researchers with extensive experience in mixed methods research design, health promotion and nutrition. A mixed‐methods design was used as a pragmatic approach. This offered a way to employ the combined strengths of quantitative and qualitative research (Tariq and Woodman 2013). Our approach included a questionnaire to gather data on individual knowledge and practices and focus groups to encourage conversation between primary caregivers about these subjects and their influences, including personal experiences and wider structural factors. This mixed‐methods convergent study design (Tariq and Woodman 2013) was used to facilitate a deep understanding of the research problem, and to reveal patterns of behaviour, knowledge and practices around oral health, and a nuanced interpretation of the experiences and social determinants influencing these patterns. The reporting of this study followed the Consolidated Criteria for Reporting Qualitative Research (COREQ) (Tong et al. 2007).

### 2.2 Recruitment and Participants

Ethical approval was granted by the university human research ethics committee, REMS No: 2021‐03012‐WALLACE.

The Australian National Oral Health plan (Australian Government Department of Health and Aged Care 2016) revealed four priority populations requiring oral health support: people experiencing social disadvantage, people in rural and remote areas, Aboriginal and Torres Strait Islander peoples and people with additional/specialised health needs. While educational attainment, Aboriginal status and geographical location of participants were captured, the fourth subgroup was not.

English language status is a known determinant of poorer oral health outcomes (Australian Government Department of Health and Aged Care 2016; Trinh et al. 2022). The high percentage of migrants residing in WA, especially the rural and remote areas selected for our study influenced the decision to also capture participants' English language status.

Purposive sampling was used to recruit participants across the Perth Metropolitan Region (Metro) (high population density administrative area of Perth and conurbation areas) and across the Great Southern Region of WA (low‐density population regional area, mainly rural). All participants were primary caregivers of children aged under 5 years at the time of data collection.

Researcher ME made initial contact with two Great Southern Region facilitators via their mutual research network. A series of online meetings were then held between these facilitator leads and the research group to discuss the study aim and processes. Following this, the facilitators advertised the study with their parent and child community early years group. The Perth Metro focus group sessions were advertised via e‐flyers using university, professional and personal social media. Interested participants were emailed a participant information letter, explaining the aim of the research and details of the focus group proceedings. Focus groups were arranged at a time and date suitable to all parties. Informed consent was gained from all participants.

### 2.3 Data Collection and Analysis

Data collection took place between February 2022 and April 2023. The questionnaire was based on relevant questions from previous studies: ‘Mothers’/guardian knowledge about promoting infant children's oral health (Akpabio et al. 2008) and ‘Parental oral health’ (Vermaire et al. 2010). All questions relating to tooth safe foods and drinks aligned with the recommendations in Australia's National Oral Health Plan 2015–2024 (Australian Government Department of Health and Aged Care 2016) and Infant Feeding Guidelines (National Health and Medical Research Council 2012). Thirteen questions and statements on diet, dental hygiene and dental visits were posed with responses of yes/no/unsure or true/false/unsure. Question guides were piloted with primary caregivers in both geographical recruitment areas and adapted for clarity and cultural appropriateness. Pilot data were not included in the results.

Ten focus groups were held where participants (*n* = 42) completed the questionnaire immediately prior to the focus group sessions. These focus groups were held online via Teams (*n* = 6) or face to face (*n* = 4). Both formats were audio‐recorded. Focus groups involving participants with English as an additional language (EAL) were held face‐to‐face. The questionnaires were administered to participants via Qualtrics prior to the online Teams focus group sessions and completed by participants as paper copies immediately prior to face‐to‐face focus group sessions, with the purpose of stimulating conversation and discussion. Members of the research team (L.A., R.W. and E.W.) facilitated all focus group sessions. Two of the face‐to‐face focus group sessions were held in a local community health centre, during a weekly parent and child community early years group session in a small town in the Great Southern Region. These sessions were supported by interpreters/connect workers who had completed focus group training provided by the research team. Focus group facilitators were not previously known to the participants but were introduced by the interpreters/connect workers at the start of each session. Each focus group lasted 40–60 min and was facilitated by one of three research team members, all experienced researchers and moderators with an interest and expertise in oral health and health promotion (L.A., R.W. and E.G.). Field notes were taken at each focus group and considered with transcribed conversations during data analysis. Recruitment continued until data saturation was reached in focus group analysis. Data were transcribed verbatim and coded collaboratively by L.A., R.W., E.G. and E.W.

Questionnaire data underwent simple descriptive statistical analysis. Findings are displayed in tables as number/total number for a group (education level, geography and language). Chi‐square analysis did not detect significant differences in quantitative data by demographic variables. The purpose of our qualitative‐led mixed methods study was to explore patterns in participant knowledge, behaviours and experiences across different social determinants, rather than infer causal relationships; hence, the inferential analysis of quantitative data by demographic variables was not included in the paper.

Focus group transcripts were entered into Nvivo v12. Data were read and re‐read by the research team. Initial codes were developed and agreed upon, which were then merged into higher order codes (Braun and Clarke 2020). Following Braun and Clarke's codebook approach (Braun and Clarke 2020), these higher order codes were then organised as themes within a predetermined codebook or coding frame as follows: tooth safe diet and feeding practices, oral hygiene and oral health services—reflecting the three aspects of the study's overall aim. These inductively developed themes offer an overview as well as a deeper understanding of participants perspectives, experiences and lived experiences around child oral health knowledge, practices experiences.

## 3 Findings

Occasionally, a participant did not respond to every questionnaire statement. Therefore, denominator totals for percentage calculations vary slightly. As three participants did not provide their educational demographic data, answers for this group are out of 39 not 42. Percentages are rounded up. Focus group themes and subthemes follow questionnaire data discussions, illustrated by participant verbatim quotes.

### 3.1 Participant Characteristics

Table A1 provides participant demographic information. This reveals a similar proportion of Perth Metro (55%) and regional (45%) participants (from a small town in the Great Southern Region of WA) and those with post‐secondary (56%) and no post‐secondary education (44%). More variation existed between those with English as a first (61%) and additional (31%) language (EAL). All participants with EAL were migrants from the Asian continent. One participant identified as an Australian Aboriginal person. Participants were all female bar one and age ranged from 21 to 47 years, with a mean age of 33 years. All participants had one or more children aged under 5 years. Just two of these children had no erupted teeth.

### 3.2 Category One: Tooth Safe Diet and Feeding Practices

Table A2 provides responses to correct statements about knowledge of drinks that are tooth safe for infant (primary) teeth when given in a bottle or sippy cup (training cup or beaker). Table A3 provides responses to correct statements on knowledge of tooth safe snack foods. Correct statements were derived from oral health recommendations from the Australia's National Oral Health Plan 2015–2024 (Australian Government Department of Health and Aged Care 2016) and Infant Feeding Guidelines (National Health and Medical Research Council 2012).

Participant responses to questions about tooth safe snack foods and drinks were mixed, revealing uncertainty. Most participants correctly identified soft drinks (89%) and sugar free soft drinks (83%) as not being tooth safe drinks. There were fewer correct responses about milk‐based drinks, and the greatest uncertainty or incorrect perceptions were reported regarding cow's milk (40%). A few participants incorrectly responded that bottled water (24%) or tap water (17%) was unsafe.

Fewer than 50% of participants correctly identified dried fruit or fruit yoghurt in pouches as not being tooth safe foods (Table A3). About 12.5% of participants were unsure if lollies (Australian term for candy or sweets) were tooth safe. The percentage of correct responses according to social determinants (geography, education level and English language) for tooth safe snacks and foods were higher across all variables for participants living in the Perth Metro area but varied for education and English language determinants.

Focus group discussions on foods and drinks reflected the responses provided in the questionnaires:

‘Probably the biggest (cause of tooth decay) would be sugary drinks … Coke and stuff like that. And probably lollies.’ (FG3)

Confusion was evident around the safety of milk and water products:

‘I feel like breast milk would be even more harm for their teeth than formula. I don't know what's in formula and cow's milk.’ (FG5)

Focus group discussions about drinks in bottles and sippy cups revealed some participant awareness of the impact of t prolonged exposure on infant teeth:

‘I guess it's just about that constant sugar on their teeth.’ (FG4)

The most common reported source of oral health information about foods and drinks suitable for children was the child health nurse, although some participants did not recall having any such discussions. Participants felt food labelling was not *a* helpful *information source*:

‘They don't make it [sugar content] clear on the labelling. If you don't do your own research, you probably don't realise how bad they actually are.’ (FG5)

Pressure from their children around certain feeding practices convinced some participants to put aside their best intentions. A parent of a boy aged 2 years explained:

‘I find it difficult to take his bottle away from him at night.’ (FG2)

The two regional focus groups mentioned the cost and availability of healthy food as a reason they sometimes chose cheaper sugar rich processed foods for their family:

‘Healthy food is more expensive (in regional areas).’ (FG9)

‘It's hard to get fresh fruit in (small hometown in regional Western Australia).’ (FG9)

There was also an uncertainty among migrant/EAL participants about how to cook and prepare healthy meals with the Australian foods available to them.

### 3.3 Category two: Oral Hygiene

Table A4 shows oral hygiene knowledge about brushing, flossing, and cleaning gums and dummies (pacifiers). Data on correct responses to statements on these topics are provided.

Confusion around recommended child oral hygiene practices was also evident. The agreement/uncertainty response from 9% of participants to the statement: ‘keeping baby teeth is not very important, after all they fall out’ was concerning. The most common misperception involved fluoridated toothpaste, with 28% of participants correctly identifying the false statement ‘fluoridated toothpaste should be used only after a child reaches four years of age’.

Focus group discussions about the recommend age to commence tooth‐brushing and how to correctly brush children's teeth was mixed:

‘I've got three children, and I would still have to look back and say, ‘what age are we supposed to be doing x, y, and z?’’ (FG1)

Most participants reported not sucking their children's dummies themselves to clean them. However, a few participants demonstrated confusion about whether or not this practice was considered harmful:

‘See, that's a new thing I keep hearing about, don't suck on your kids dummy. I've never known about this before.’ (FG4)

Oral hygiene knowledge was higher among Perth Metro participants and those with post school education but for participants with English as a first or additional language, the results were less certain. Participants discussed the source of their oral hygiene knowledge, with most citing the child health nurse as the primary source of this information. Some participants, however, stated that oral health did not seem a priority for the child health nurse:

‘I don't feel there's much emphasis on dental health in preschool kids.’ (FG1)

Others qualified this sentiment, stating it may be due to the many competing health messages child health nurses had to convey:

‘The child health nurse is covering many things.’ (FG1)

Several participants referred to their dentist as a source of face‐to‐face information. Just two participants accessed oral health information from their general practitioner. Informal knowledge from family was not seen as helpful or on reflection, correct:

‘My parents didn't know anything either and it (brushing) just didn't get passed to us.’ (FG9)

Table A5 shows responses to statements about fluoride in general. Many participants had little knowledge about the benefits of fluoride for their children's teeth:

‘I don't know anything about that (fluoride in tap water).’ (FG6)

Discussing her child aged 2 years one participant stated:

‘I was worried about him having too much … I don't know what it does though, like what is the issue with that?’ (FG2)

Participants also identified a lack of consistency in the information available on the topic of fluoride:

‘There's really conflicting information, especially for young kids in relation to fluoride and when to start giving kids fluoride.’ (FG10)

Nearly half of participants incorrectly responded that bottled water was a good source of fluoride.

**FIGURE 2:**
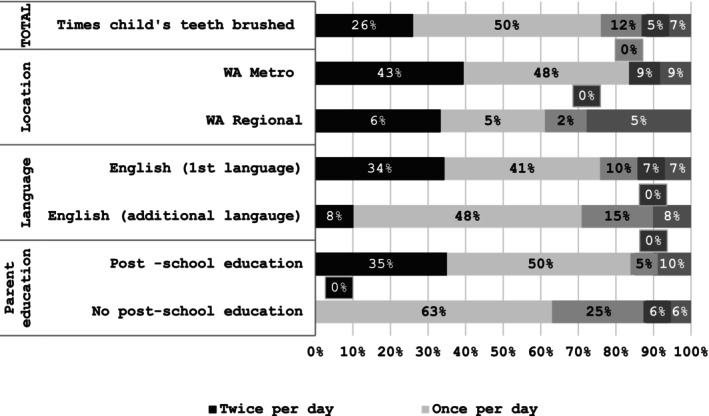
Tooth brushing practice. *Note:* Correct response is once per day. *Twice a day and>Twice a day category excluded from graph as both were 0%.

**FIGURE 3:**
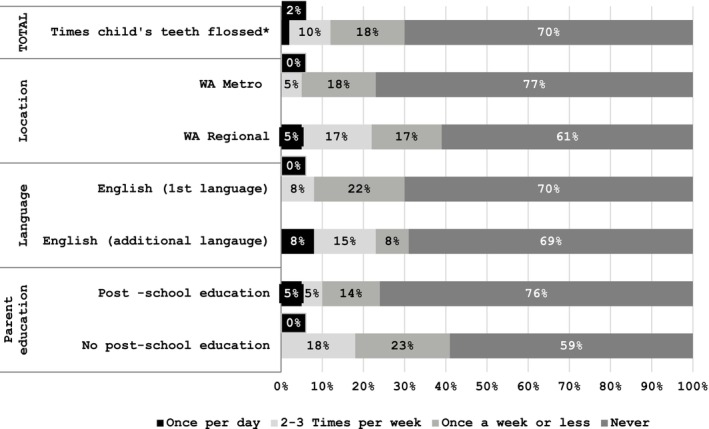
Flossing practices.

Oral hygiene practice questionnaire responses (brushing and flossing children's teeth) are shown in Figure 2 and Figure 3. Just 26% of participants reported they brushed their children's teeth twice a day as recommended (Tariq and Woodman 2013), while 14% reported they brushed their children's teeth less than twice a day or never. The age or stage of development that tooth brushing with a child commenced varied. Some parents started brushing once a tooth erupted:

‘As soon as their teeth popped up, we started brushing.’ (FG4)

Other parents started brushing when their child was a toddler.

‘It really wasn't until he was a bit older that I could really put it into the evening routine. I'd say, like, 18 months to two years that's when it became pretty standard.’ (FG1)

Flossing was rarely practiced, with 70% of participants reporting they never flossed their children's teeth and most participants unaware they should be doing this regularly.

‘I don't think I've ever really (been told) it's not really been on the radar.’ (FG4)

Focus group conversations expanded on the factors that influenced these practices. The reasons participants gave for not brushing their children's teeth twice a day were most often associated with time and prioritisation of other tasks rather than a lack of knowledge.

‘The mindset that baby teeth are not permanent so ‘that [toothbrushing] priority is really low on my list of things to do.’ (FG6)

‘We do it at night and not always in the morning. Depends on if we are in a rush.’ (FG 3)

The two participants who stated they did floss their children's teeth revealed they began after their child developed a tooth cavity:

‘He flosses (son aged 4) … he's got teeth issues, so were, like, on it.’ (FG6)

Brushing their children's teeth the recommended twice a day was markedly higher in participants from the Perth Metro area, who were post school educated and had English as a first language. Reflecting the discussions about giving children sugary drinks, snacks and bottles at night, some participants disclosed a desire to avoid conflict over the issue of brushing:

‘The last thing you want is a fight on your hands.’ (FG8)

Primary caregivers of older infants and children commonly raised the issue of their child's independence:

‘You know, there's only so much that you can force upon a child once they get to a certain age. It's probably my biggest challenge.’ (FG6)

This more relaxed attitude was less common among participants with English as an additional language and those from regional communities, who tended to be more certain about taking charge:

‘I make him brush; he is developing the habit.’ (FG9)

### 3.4 Category Three: Dental Service Engagement

Table A6 presents data about participants' knowledge of dental service engagement (engagement with dental health professionals regarding their infant's and children's teeth). A key finding is that almost a quarter (23%) of all participants agreed with or were unsure about the incorrect statement, ‘Cavities in baby teeth don't matter since they fall out anyway.’

**FIGURE 4:**
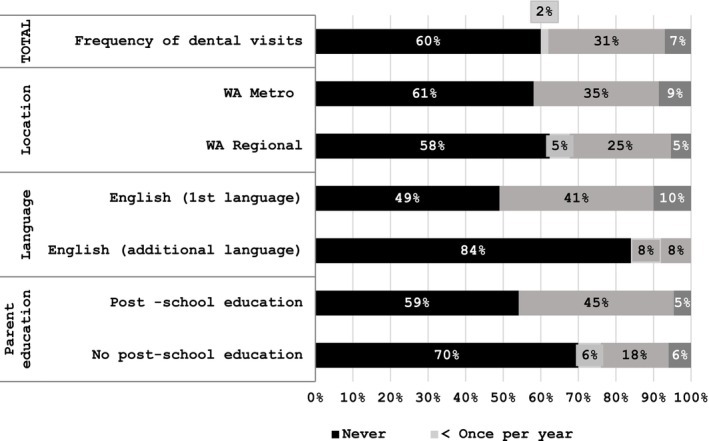
Dental health engagement practices. Major treatment and emergency categories excluded from graph as both were 0%.

**FIGURE 5:**
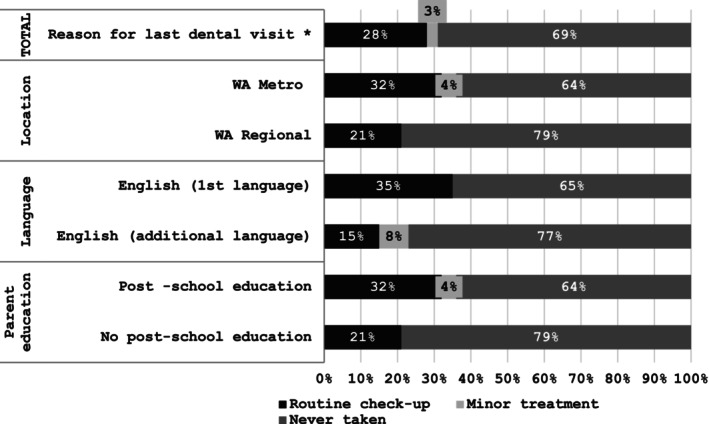
Dental service engagement reasons.

Figure 4 reveals dental health engagement practices. Despite 66% of participants agreeing that children should visit their dentist six‐monthly, fewer did so in practice, with two‐thirds having never taken their child to a dentist. Figure 5 reveals reasons given for dental service engagement. The participants who had taken their child to see a dentist had done so for a check‐up, indicating some preventative engagement.

The focus groups revealed several factors that influenced participants' engagement with dental services for their children. Overall, many felt they lacked knowledge:

‘I don't know. How soon do you take them to the dentist? Is it as soon as their teeth come?’ (FG7)

Some parents thought children did not need to see a dentist until around 5 years of age:

‘So, my oldest is 4 [years], so I haven't taken her yet [to dentist,] but I sort of thought maybe when she turns 5 [years] that I would take her for the first time.’ (FG3)

Like flossing, consultation with a dental practitioner sometimes began after a dental problem was discovered:

‘What prompted me was that I saw plaque build‐up on his teeth and so I thought, ‘Oh my god I have to take him to the dentist.’ (FG9)

Participants' own perceptions of dentists also influenced their practices. A few avoided the dentist because they expected to be chastised about their parenting, rather than supported:

‘I haven't gone because I don't want a grilling from the dentist … they will just tell me to get rid of the dummy but no advice on how to do it.’ (FG10)

There was a common perception that their dentist was not child focused. One participant explained they delayed their child's first visit because of this:

‘I just knew they wouldn't be comfortable to sit in the chair under the age of four. I think they would have been scared [of the dentist].’ (FG7)

Others, however, described how their dentist tried to cater for the needs of young children:

‘Yeah, mine is good. They were like ‘we will get him in a booster seat’, there is a choice of different prints on the booster seats, TV on the roof.’ (FG8)

Several participants reported their own fear about visiting a dentist influenced their decisions not to take their child:

‘I always had this anxiety about going to the dentist. [Made me] Probably less likely [to take child to the dentist]. It's probably in the back of my mind.’ (FG8)

The costs associated with a child's dental care was commonly identified as a barrier to access. Private medical insurance was often described as essential:

‘Cost for me is a major thing because if I didn't have [private health insurance] … there's no way I would have been able to afford [it].’ (FG2)

The cost of dental care was also indicated as a challenge to access for many participants with EAL:

‘Sometimes cost does affect me. I mean my husband still learning English so he can't really get a job that easily.’ (FG8)

A few participants in the regional focus groups were aware of a free public dental service, but that this service was not local and had a long waiting list:

‘Free public dentist in [Town A) ‐ that is an hour's drive or there is another one in [Town B] that is 2 hours away.’ (FG8)

Few participants had heard of the Child Dental Benefits Schedule, which is a government means tested dental financial support scheme, and those who had did not understand how the scheme worked:

‘Yeah, I have. I do not understand it though. It's weird ‐ the dental nurse asked me to sign this bit of paper and I have no idea.’ (FG6)

## 4 Discussion and Recommendations

An adaptation of the Fisher‐Owens et al. (2007) socioecological model of child oral health is applied to the findings. Each category of findings is interpreted across the model's dimensions and against published literature. Our study's focus on preschool children prevents the separation of child and family level dimensions of influence because of the child's dependence on primary caregivers. These dimensions of the model have therefore been addressed together. Recommendations for health practice, health service delivery and wider oral health policy (Fisher‐Owens et al. 2007) are drawn from this discussion (Table A7).

### 4.1 Family and Child: The Need for Consistent and Contemporary Evidence‐Based Child Oral Health Promotion Messages

What is apparent from our study is the general confusion among families from all social backgrounds about many areas of child oral health, and the need for accessible, universal and consistent health promotion advice and services.

On a positive note, the findings revealed a sound knowledge about the harms of obviously sugar‐rich products, such as lollies and sugar‐sweetened beverages (SSBs) and general awareness of the harms associated with sugar‐free drinks and fruit juices. This finding concurs with an earlier study of parents in Victoria, Australia (Virgo‐Milton et al. 2016). Our study also found however that participants were less sure of the ‘tooth‐friendliness’ of milks and some snack foods commonly marketed to children. This inconsistency means primary caregivers may not be able to make the safest choices of foods and drinks to protect their children's teeth.

Focus group discussions also revealed some understanding of the harms prolonged exposure to some foods and drinks through sucking; however, most also indicated a belief that fruit yoghurt pouches, which rely on this feeding mechanism, and may include free sugars, were tooth safe. This contradictory finding, plus the confusion about the ‘tooth‐friendliness’ of milk suggests a lack of awareness about the different types of sugars (free and naturally occurring) and their impact on tooth health. Free sugars are the most detrimental to a child's oral and overall health (Food Standards Australia and New Zealand 2019). Soft drinks including sodas, fruit juice and cordial are the most common source of free sugars in the diet (Food Standards Australia and New Zealand 2019). The World Health Organisation define free sugars as ‘monosaccharides and disaccharides added to foods and beverages by the manufacturer, cook or consumer, and sugars naturally present in honey, syrups, fruit juices and fruit juice concentrates’ (World Health Organization 2015, 4). Naturally occurring sugars (found in milks, unsweetened yoghurt and whole, fresh fruit) are less harmful to oral and overall health (Moynihan 2016). Indeed, lactose (the naturally occurring sugar found in milk and other dairy products) is considered the ‘least cariogenic of all dietary, fermentable carbohydrates’ (Shkembi and Huppertz 2023, 1470). Given that milk and unsweetened yoghurt are an excellent source of calcium and protein, essential for child bone and tooth health (Li et al. 2023), caregivers who are unclear about the tooth safety of dairy foods (when consumed appropriately) could inadvertently be increasing their child's risk of ECC by avoiding them and by substituting them with more harmful foods and drinks (Li et al. 2023). To redress this, advice and information on free and added sugars and appropriate feeding practices is needed on food and drinks labels, and caregivers may need support on interpreting this information.

Participant knowledge of oral hygiene behaviours was similarly confused. While the questionnaire demonstrated some awareness of correct oral hygiene regimes, it also revealed participants did not necessarily adhere to these practices, particularly regular tooth brushing. The idea of flossing children's teeth as a preventative measure was almost completely unconsidered, evident only in families where children had existing tooth problems. The Australian Dental Association (Australian Dental Association [ADA] 2024) suggest that flossing is important for children once they have two teeth touching side by side, although there appear to be no government guidelines to this effect. Furthermore, there is no strong evidence about the impact of flossing on dental caries and a systematic review (de Oliveira et al. 2017) reported only one study evidencing the positive impact of flossing primary teeth on dental caries. It is nonetheless a positive oral health behaviour, so should not be discouraged, as healthy oral habits embedded in childhood will continue into adulthood, especially pertinent as a national study of Australian adults indicated only 55% of respondents flossed regularly (Luzzi et al. 2020).

In our study, discussions about fluoride revealed considerable confusion, not surprising given the conflicting information available. Participants demonstrated poor understanding of the benefits of fluoride, through its presence in toothpaste and water, despite water fluoridation and fluoride toothpaste being widely acknowledged as a successful strategy to prevent dental caries (Spencer 2006; Do 2020). Recommendations for brushing children's teeth include age of commencement, amount of toothpaste applied to a brush, the size of the working head of the brush, spitting out toothpaste foam and not rinsing, and not eating or licking toothpaste directly from the toothpaste tube [and] the availability and recommended use of a low fluoride children's toothpaste (Do 2020). This report notes that most parents brush their children's teeth in line with guidelines, aside from approximately 30% of children when brushing with fluoridated toothpaste began before the recommended age of 18 months. About a quarter of our participants thought bottled water was ‘tooth safe’, mirroring the National Oral Health Survey for children and young people aged up to 18 years that reported just half of parents knew tap water was better for teeth than bottled water because of the added fluoride (Rhodes 2018). Such uncertainties among caregivers may have been influenced by inconsistent messaging and oral health misinformation across media sources participants accessed for oral health advice, a phenomenon previously described by Ruiz et al. (2022). Although national guidelines on the use of fluoride (Spencer 2006; Do 2020) provide clear oral health advice to health professionals this information needs to be carefully framed for caregiver consumption to ensure that health inequities are not perpetuated through ‘victim blaming’. Suggesting poor oral health is solely an individual responsibility can have unintended negative consequences, including on mental health (Nguyen and Lin 2023).

### 4.2 Community Influences, Services and Relationships: The Need for Accessible Sources of Oral Health Advice and Support

The ADA (2023) framework (Australian Dental Association 2023) is an appropriate basis for oral health education and promotion. This document, intended to ‘serve as a framework for health practitioners and health promoters’ (Australian Dental Association 2023, 5), includes consistent messages on some of the main areas of confusion identified in our study, including advice on putting children and babies to bed with a bottle, fluoridation of water, tooth brushing regimes, use of fluoridated toothpaste and dental check‐up regimes. Our study indicates there are additional topics that should be addressed including information about local child‐friendly dental services, and government dental financial support, such as the Child Dental Benefit Schedule, currently under‐utilised in WA (Putri et al. 2020).

Another topic that appears important to child oral health is the guidance provided by parents to their children in relation to tooth‐brushing, as recommendations suggest parents should assist their children with tooth‐brushing until the age of 8–9 years (Australian Dental Association 2023). The more relaxed approach described by some participants that allowed very young children to brush their own teeth, and acquiescing to children who refuse to brush their teeth was described in an earlier study of Australian mothers in Victoria (Virgo‐Milton et al. 2016). Health promotion strategies to address dental hygiene practices should include practical advice and support for caregivers so that correct, regular, and supervised brushing can be achieved with their children.

Poor health literacy is associated with worse health outcomes (Australian Institute of Health and Welfare 2023b). Given 40% of people may not be able to comprehend health information written for them (Gursul 2022), it is important to consider alternative means of dissemination. Timm et al. (2022), for example, advise strategies to promote parental health literacy to support young children's health include involving the whole family, applying home‐based activities and offering flexible models of information delivery.

### 4.3 Wider Sociocultural Influences: Collaborative Approaches Are Required to Redress Inequity in Child Oral Health Services

The guiding principles of the National Oral Health Plan include a population health approach based on proportionate universalism (Australian Government Department of Health and Aged Care 2016). This strategy translates to accessible dental services for all Australians, dental care within a clinically acceptable timeframe and the capacity of all health care professionals to provide relevant and accurate oral health information. Our study shows current WA dental health services for preschool children tend not to reflect these principles, with accessibility to services influenced by cost, geography and a community's cultural background.

The cost of dental care, both preventative and treatment‐based, was a major barrier for many participants in our study. This situation, contrary to the aforementioned principle of proportionate universalism, can present a significant obstacle to achieving equitable oral health outcomes for Australian children (Australian Government Department of Health and Aged Care 2016). Although the Child Dental Benefit Schedule offers financial support for some families, these scheme is under‐utilised (Putri et al. 2020), offers only basic dental services and is available only for families in receipt of certain Centrelink payments (Australian Government 2024).

It is acknowledged that the social determinant of geography is an important factor influencing oral health for Australians living in regional and remote areas of the country, often due to lower availability of primary healthcare services (Thomas et al. 2014). While answers to this complex issue are outside the article's scope, it is apparent a flexible approach is required (Thomas et al. 2014). Tele dentistry has been evaluated positively in Australia (Irving et al. 2018) and is an approach that may supplement dental services and oral health promotion programmes in underserved populations.

While our study did not explore in depth cultural differences—such as historical and political influences on cultural oral health practices—it did reveal some important influences on the capacity of participants to access and follow oral health advice. One issue involved tooth‐safe, healthy food acquisition and preparation for participants from EAL backgrounds. This finding draws parallels with an international review (Riggs et al. 2019) that found migrants with EAL were unsure how to cook and prepare tooth safe foods, a factor that may adversely influence their capacity to ensure their children had a tooth safe diet.

Oral health service planning and delivery requires a ‘collaborative coordinated approach’ to empower Australians to take control of their family's oral health (Australian Government Department of Health and Aged Care 2016). Such collaborative approaches should address time capacity issues for child health nurses that may restrict the prioritisation of oral health promotion activities and enable consistent oral health messages to be delivered to families. Representation from stakeholders with lived experience of wider social‐cultural factors important to child oral health is also needed in any collaboration process (Veale et al. 2016). Findings from our study indicate this should include contact workers, translators and health workers from the same cultural background as the community (Marcus et al. 2022) and although not addressed in our study, Aboriginal Health Worker involvement (Butten et al. 2019).

## 5 Strengths and Limitations

The sample sizes were relatively small, limiting generalisability, and did not explore demographics such as Aboriginal background. Moreover, the reliability and validity of the data collection instruments were not subject to statistical scrutiny and some survey questions could be considered leading, potentially biasing the results. Our study was however, the first in WA to explore primary caregivers' experiences and understanding of child oral health. The study provides authentic, contextualised insights into child oral health, on which further research and WA oral health promotion strategies can be built.

## 6 Conclusions

The exploration of WA primary caregivers' child oral health knowledge, experiences and practices has revealed that despite the availability of child oral health best‐practice guidelines and consensus statements, WA primary caregivers are confused and poorly equipped to promote their children's oral health, taking a reactive approach to dental service engagement. This situation is compounded by current Government dental service funding policy for preschool children, food labelling policy, and inequity in dental service availability. Recommendations include consistent, collaborative oral health promotion education, and equitable and accessible dental health services for preschool children.

## Author Contributions

**Lesley Andrew:** conceptualization, investigation, funding acquisition, writing – original draft, methodology, validation, writing – review and editing, project administration, data curation, formal analysis. **Elizabeth Wenden:** formal analysis, writing – review and editing. **Mohamed Estai:** conceptualization, investigation, funding acquisition, methodology, validation, writing – review and editing. **Ruth Wallace:** conceptualization, investigation, funding acquisition, methodology, validation, writing – review and editing, project administration, data curation.

## Consent

The authors have nothing to report.

## Conflicts of Interest

The authors declare no conflicts of interest.

## Data Availability

The quantitative data that support the findings of this study are available on request from the corresponding author. The data are not publicly available due to privacy or ethical restrictions.

## Appendix

**TABLE A1:** Participant demographics.

Participant demographics (*n* = 42)		*n* (%)
Level of education (missing = 3)	Secondary school	17 (43.5)
	Post‐secondary school	22 (56.4)
Location	WA Perth metropolitan area	23 (55.0)
	WA regional areas	19 (45.0)
English speaking	First language	29 (69.0)
	Additional language	13 (31.0)

Abbreviation: WA, Western Australia.

**TABLE A2:** Primary caregiver knowledge of tooth safe drinks.

Drink type	Total correct	Geographical location		Main language		Primary caregiver education	
		WA metro	WA regional	English (1st language)	English (additional language)	Post‐school education	No post‐school education
	*n* (%)	*n* (%)	*n* (%)	*n* (%)	*n* (%)	*n* (%)	*n* (%)
Tap water (T)	34 (83)	19 (86)	15 (79)	25 (89)	9 (69)	19 (86)	12 (75)
Bottled water (T)	31 (76)	15 (68)	16 (84)	20 (71)	11 (85)	17 (85)	13 (77)
Cow's milk (T)	24 (60)	11 (52)	13 (79)	15 (56)	9 (69)	14 (64)	9 (53)
Breast milk (T)	35 (88)	20 (95)	15 (79)	24 (89)	11 (85)	22 (100)	11 (65)
Formula milk (T)	25 (63)	10 (48)	15 (79)	18 (67)	7 (54)	12 (55)	11 (65)
Fruit cordial (diluted) (F)	33 (80)	19 (83)	14 (74)	23 (79)	9 (69)	21 (95)	10 (59)
Fruit juice/fruit juice drinks (F)	33 (80)	23 (100)	10 (56)	22 (79)	9 (69)	20 (91)	11 (59)
Full sugar soft drinks, e.g., Cola. (F)	37 (93)	21 (100)	16 (84)	25 (93)	12 (92)	20 (91)	15 (88)
Energy drinks e.g., Red Bull (F)	36 (92)	20 (100)	15 (79)	27 (96)	9 (82)	17 (100)	10 (83)
Sugar‐free soft drinks. e.g., Pepsi Max (F)	35 (88)	20 (95)	15 (79)	23 (27)	12 (92)	19 (90)	15 (88)

Abbreviations: (F) – False; (T) – True.

**TABLE A3:** Primary caregiver knowledge of tooth safe snacks.

Food type	Total correct	Geographical location		Language		Primary caregiver education	
		Perth metro	WA regional	English (1st language)	English (additional language)	Post‐school education	No post‐school education
	*n* (%)	*n* (%)	*n* (%)	*n* (%)	*n* (%)	*n* (%)	*n* (%)
Dried fruit (e.g., sultanas, banana pieces) (F)	20 (48)	17 (74)	3 (16)	17 (59)	3 (23)	14 (64)	5 (29)
Fruit yoghurt in pouches (F)	10 (24)	8 (35)	2 (11)	4 (14)	6 (46)	2 (11)	6 (27)
Fruit lollies/candy/sweets, e.g., fruit straps (F)	37 (88)	23 (100)	14 (74)	25 (86)	12 (92)	22 (100)	10 (59)

Abbreviations: (F) – False; (T) – True.

**TABLE A4:** Oral hygiene knowledge.

Statement	Total correct	Geographical location		Language		Primary caregiver education	
		Perth metro	WA regional	English (1st language)	English (additional language)	Post‐school education	No post‐school education
	*n* (%)	*n* (%)	*n* (%)	*n* (%)	*n* (%)	*n* (%)	*n* (%)
Keeping baby teeth clean is not very important; after all, they fall out (F)	32 (91)	18 (90)	14 (74)	20 (87)	12 (100)	19 (100)	13 (76)
The right time to replace a child's toothbrush is at least every 6 months (T)	23 (58)	18 (90)	5 (26)	19 (73)	4 (31)	15 (68)	4 (31)
Children do not really need their own toothbrush until all their teeth have arrived (F)	25 (75)	15 (71)	10 (53)	16 (64)	9 (69)	15 (75)	9 (69)
I do not need to brush my child's teeth every day until they get their permanent teeth (F)	30 (83)	17 (100)	13 (68)	20 (91)	10 (77)	18 (86)	10 (77)
Before a baby has their first tooth, parents/carers should clean the baby's gums with a cloth (T)	27 (69)	15 (71)	12 (67)	15 (58)	12 (92)	17 (77)	12 (92)
A pea size amount of toothpaste is all that is needed to brush a child's teeth (T)	31 (79)	16 (80)	15 (79)	22 (85)	9 (69)	18 (82)	9 (69)
Fluoridated toothpaste should be used only after a child reaches 4 years of age (F)	3 (13)	4 (22)	4 (19)	4 (15)	4 (31)	4 (17)	4 (31)
It is safe for my child's teeth if I clean their dummy by sucking on it (F)	31 (82)	17 (89)	14 (74)	25 (100)	6 (46)	20 (87)	6 (46)

(T) – True.

(F) ‐ False.

**TABLE A5:** Parental perceptions of fluoride.

Statement	TOTAL correct	Geographical location		Language		Primary caregiver education	
		Perth Metro	WA Regional	English (1st language)	English (additional language)	Post‐school education	No post‐school education
	*n* (%)	*n* (%)	*n* (%)	*n* (%)	*n* (%)	*n* (%)	*n* (%)
Tap water is a good source of fluoride for my child (T)	12 (31)	11 (55)	12 (63)	15 (58)	8 (62)	14 (64)	9 (53)
Bottled water is a good source of fluoride for my child (F)	19 (49)	12 (60)	2 (11)	12 (46)	2 (15)	12 (55)	2 (12)
Fluoridated water is important to prevent child dental caries (T)	14 (36)	12 (65)	6 (32)	16 (62)	3 (23)	13 (59)	9 (35)

(T) = True; (F) = False.

**TABLE A6:** Dental service engagement knowledge.

Statement	Total correct	Geographical location		Language		Primary caregiver education	
		Perth metro	WA regional	English (1st language)	English (additional language)	Post‐school education	No post‐school education
	*n* (%)	*n* (%)	*n* (%)	*n* (%)	*n* (%)	*n* (%)	*n* (%)
Once a child is aged 2 years, they should visit a dentist (F)	7 (17)	4 (17)	3 (16)	4 (14)	3 (23)	5 (23)	1 (6)
Children should see a dentist for a check‐up twice a year (T)	26 (63)	17 (74)	9 (50)	17 (61)	9 (69)	18 (81)	8 (47)
Cavities in baby teeth do not matter since they fall out anyway (F)	27 (77)	13 (76)	14 (78)	17 (77)	10 (77)	19 (100)	8 (50)

Abbreviations: (F) – False; (T) – True.

**TABLE A7:** Discussion and recommendations.

Categories	Family and child influences	Community service and relationship influences	Wider socio political, cultural influences	Recommendations to health practice, services and policy
Oral health promoting diet and feeding practices	Primary caregiver child oral health knowledge and understanding. Parental feeding practices ‐ for example, night‐time bottles.	Inconsistent or unclear oral health advice. Priority of oral health advice by health professionals.	Cost of and access to healthy foods. Cooking and knowledge of Australian foods for EAL/migrant families. Tooth safe food and drink labelling. Communication of health information.	Training of all primary health care staff in shared, evidence‐based oral health messages. Prioritisation of consistent messages about tooth safe food and drinks across primary health care practitioners. Inclusion of oral health promotion education in nursing degrees. Australia‐wide policy development regarding tooth‐safe food and drink labelling. Delivery of health promotion activities regarding low‐cost healthy food and its preparation. and health promotion strategies to support interpretation of tooth‐safe information.
Oral hygiene	Caregiver knowledge of brushing and flossing. Confusion around fluoride in toothpaste and drinks. Parental practices around regular tooth brushing and child reluctance. Child autonomy beliefs of caregivers. Family routines, compounding time pressures.	Inconsistent or unclear oral health advice. Priority of oral health advice by health professionals.	Social media conflicting messages on fluoride. Cultural differences regarding parenting approaches towards challenging situations with their children (brushing regimes and giving snacks and drinks).	Training of all primary health care staff in shared, evidence‐based oral health messages. Prioritisation of consistent messages about tooth safe food and drink across primary health care practitioners. Development of clear, consistent and accessible messages about fluoride in water and toothpaste. Realistic and practical parenting support and advice (brushing, introducing healthy foods, weaning off bottles etc.).
Dental service engagement	Knowledge of recommended screening/checkup dates for pre‐school children. Family income and insurance status. Caregiver perceptions and experiences of dentist. Knowledge of Child Dental Benefits Schedule	Availability and accessibility of local dental service. Availability of local child‐friendly dental services.	Cost of regular‐ proactive dental visits (check‐ups) and treatment. Lack of clarity around these costs.	Universal public dental service for children aged under 5 years based on principles of primary health care. Collaboration of local primary health care providers and dental services to ensure consistent and transparent advice and referral pathways into dental care. Primary health care, oral health and child‐friendly practices embedded in dentist degree education. Adoption of flexible dental services in regional and rural areas, such as telehealth and mobile services.
